# Cobalamin Status Among Patients with Fetal Alcohol Spectrum Disorder (FASD)—A Preliminary Study

**DOI:** 10.3390/nu17030409

**Published:** 2025-01-23

**Authors:** Magdalena Król-Dykas, Katarzyna Dyląg, Katarzyna Przybyszewska, Katarzyna Burkot, Aleksandra Tokarz, Katarzyna Kowalska, Paulina Dumnicka, Magdalena Kurnik-Łucka, Marta Zarzycka, Krzysztof Gil

**Affiliations:** 1 Department of Pathophysiology, Jagiellonian University Medical College, 31-121 Krakow, Poland; 2 St. Louis Children Hospital, 31-503 Krakow, Poland; 3 Department of Medical Biochemistry, Jagiellonian University Medical College, 31-034 Krakow, Poland

**Keywords:** fetal alcohol spectrum disorder, prenatal alcohol exposure, cobalamin, methyl donor-enriched diet, neurodevelopmental disorders, vitamin B12

## Abstract

**Background/Objectives:** Fetal alcohol spectrum disorder comprise a range of neurodevelopmental disorder caused by prenatal alcohol exposure. Recent investigations have revealed that among patients with neurodevelopmental disorders, serum cobalamin (vitamin B12) levels are substantially higher than those of healthy controls. Patients with fetal alcohol spectrum disorder similarly present with higher levels of cobalamin, yet the significance of cobalamin in the pathogenesis of fetal alcohol spectrum disorder remains to be established. This study aimed to examine cobalamin and other cobalamin status markers in patients with fetal alcohol spectrum disorder in comparison with healthy controls. **Methods:** In total, 80 patients were enrolled in the study—41 diagnosed with fetal alcohol spectrum disorder and 39 healthy controls. The diet history method was used to assess vitamin B12 intake for three days preceding blood sampling. Total vitamin B12 (cobalamin), holotranscobalamin, methylmalonic acid and soluble transcobalamin receptor (CD320) were measured in serum samples. **Results:** The daily intake of vitamin B12 was higher in patients with fetal alcohol spectrum disorder compared to controls, both in the simple analysis and after adjusting for age (OR for patients with FASD: 1.82; 95% CI: 1.16–2.87). An elevated serum cobalamin level was noted in some patients from the group with fetal alcohol spectrum disorder. No statistically significant differences were found between the groups in serum levels of cobalamin, holotranscobalamin, CD320 or methylmalonic acid. However, the correlations between cobalamin and its metabolites differed in fetal alcohol spectrum disorder as compared to those in the control group. **Conclusions:** Our study did not find any deficits of vitamin B12 and its metabolites in patients with fetal alcohol spectrum disorder. Further studies to investigate the role of vitamin B12 in the pathogenesis of fetal alcohol spectrum disorder should be established given the fact that both high and low levels of vitamin B12 may have negative health impacts.

## 1. Introduction

Fetal alcohol spectrum disorder (FASD) is an umbrella term for a group of neurodevelopmental disorders (NDs) that result from prenatal alcohol exposure (PAE) [1]. FASD is the only preventable developmental disability [2]. The mechanisms of alcohol-induced alterations to the fetal nervous system remain incompletely understood [3]. So far, it has been established that neuronal myelination is impaired in this group of patients [4,5,6,7,8].

Vitamin B12, also known as cobalamin, refers to a group of compounds characterized by a corrin ring structure with a central cobalt atom, and belongs to the class of the natural tetrapyrroles [9]. This vitamin serves as a cofactor in two reactions: the regeneration of methionine from homocysteine, which is essential for methylation and DNA synthesis, and the conversion of methylmalonyl-coenzyme A (CoA) to succinyl-CoA, an intermediate in the citric acid cycle [10]. Methylmalonic acid, an organic acid with neurotoxic properties in vitro, accumulates due to cobalamin deficiency. De novo DNA synthesis and methylation, which are essential for fast cell division and growth, especially in the growing fetus and its brain, fully depend on cobalamin [11]. Considering possible implications for cognitive development and general cognitive performance, cobalamin is crucial for neural myelination, synaptogenesis, myelination and neurotransmitter production [12]. These functions can be hampered by low cobalamin levels and can also cause brain atrophy and neurological damage [13]. On the other hand, Hope et al. in 2020 observed an increased concentration of cobalamin in individuals with NDs as compared to healthy controls [14,15]. Patients with NDs were 4.1 times more likely to have higher cobalamin levels, whereas low cobalamin levels were less common. According to the authors, these results may suggest that cobalamin should play a significant role in the pathogenesis of NDs in children, adolescents and adults. Moreover, elevated cobalamin levels appear to be clinically significant as a predictor of serious consequences in a variety of mental and developmental conditions [14]. Recently, Dylag et al. reported increased cobalamin levels among patients with FASD [16]; however, the results were confronted with population norms only. The role of cobalamin in FASD pathogenesis has not been determined yet. Thus, the aim of this study was to investigate serum levels of cobalamin and the laboratory markers of cobalamin status among patients with FASD.

## 2. Materials and Methods

### 2.1. Study Design and Participants

The study was conducted according to the Declaration of Helsinki. The study protocol gained the approval of the Bioethical Committee of Jagiellonian University (no. 1072.6120.65.2023 issued on 14 June 2023). Informed consent was obtained from all subjects involved in the study. Informed consent was obtained from the parent/caregiver and assent was obtained from patients 13 years and older.

The study was conducted in the years 2023–2024. The studied group included patients with FASD diagnosed with the Polish criteria [17], who were children and adolescents at the time of enrollment, recruited through the FASD Diagnostic Centre of St. Louis Children’s Hospital in Kraków, Poland. According to the Polish guidelines, each patient has to be evaluated by a physician and a psychologist. Based on dysmorphic features examination and neuropsychological evaluation, three diagnoses are made: fetal alcohol syndrome (FAS), neurodevelopmental disorder with prenatal alcohol exposure (ND-PAE) or “risk group for FASD”. For each participant, the diagnosis was made prior to the start of the project. The exclusion criteria were the following: age under 1 years, pregnancy, diagnosed condition characterized by malabsorption (information obtained from medical history or medical records about a child’s diagnosis of diseases associated with impaired absorption of nutrients, with a particular focus on diseases that may impair vitamin B12 absorption, e.g., Addison–Biermer anemia or celiac disease; we adopted this criterion as an exclusion criterion from participation in the study to avoid the impact of malabsorption disorders on the obtained laboratory results of vitamin B12 blood concentrations), vitamin B12 supplementation one month before inclusion in the study (based on the medical history given by caregivers), and enteral or parenteral nutrition.

The control group was recruited from the patients of St. Louis Children’s Hospital in Kraków and included children and adolescents without chronic diseases admitted to the hospital for emergency reasons such as respiratory infections, acute diarrhea, fainting, etc. Participation in the study was offered after recovery. By design, the control group included healthy children and adolescents with typical development. The exclusion criteria were the same as for the studied group; moreover, we excluded children and adolescents with identified autism spectrum disorder; attention deficit hyperactivity disorder (ADHD); intellectual disability; presence of the features indicative of possible previously unrecognized FASD, such as growth deficiency and/or low body weight, microcephaly or dysmorphic features; and information on prenatal alcohol exposure in medical history.

### 2.2. The Assessment of Daily Vitamin B12 Intake

After obtaining consent for participation in the study, the diet history method was used to estimate the amount of vitamin B12 intake during the period prior to participation in the study [18]. The only vitamin B12 source was the patients’ diet; patients on oral or parenteral supplementation were excluded from the study. Full dietary information from 3 previous and typical days prior to participation in the study was obtained to quantitatively assess the mean vitamin B12 intake. The method of the 3-day dietary record was chosen to provide both good recall and adequate information to count the mean intake [19]. All participants received a hard copy, single-page printout of a standard 3-day food diary to document their dietary intake. Participants on the 3-day food diary were instructed to document and assess the specifics of all meals and snacks taken, including portion sizes [20]. The 3-day dietary record is widely used in clinical practice and is considered a method of reference in the validation of other tools [21,22]. Participants had the choice to submit the diary in person at the hospital or to scan and send their 3-day food diary to the study email address. All 3-day food diaries were encoded for confidentiality; those sent via email were promptly printed, and the email was erased to preserve confidentiality. Then, the patient’s estimated vitamin daily B12 intake was calculated [23]. Tables of the estimated cobalamin concentrations in specific products were used to estimate daily intake [24].

### 2.3. Laboratory Tests

Fasting blood samples (5 mL) were collected from the study participants after a night’s rest. Blood was collected in serum tubes with clot activator and serum separator (Vacuette, Greiner Bio-One, Kremsmünster, Austria), centrifuged within 30 min of collection and the resulting serum was divided into aliquots for further laboratory testing. One aliquot was used to measure total serum B12 concentration with an automated assay. These measurements were performed in a routine medical laboratory on the day of blood collection. The other aliquots were immediately frozen in −20 °C for further laboratory testing performed in series using commercially available enzyme-linked immunosorbent assays using the Alinity analyser (Abbott Laboratories, Chicago, IL, US) (ELISAs- ELK9024, Human CNCbl(Cyanocobalamin) ELISA kit, 96T).

The following laboratory measurements were performed:

(1) Total vitamin B12 (total cobalamin) was measured with a chemiluminescent microparticle immunoassay using an automated analyzer (Alinity, Abbott Laboratories, Chicago, IL, USA). The laboratory method used for the measurements was certified for in vitro diagnostics and underwent standard medical laboratory internal and external quality control. Age- and sex-specific reference intervals were used to interpret the results of the test according to the study of Bailey et al. [25], i.e., 209–1190 pmol/L in patients aged 1 year to 9 years, 186–830 pmol/L in patients aged 9 to 13 years, 180–655 pmol/L in adolescents aged 14 to 17 years and 150–599 pmol/L in adolescents aged 17 and 18 years;
(2) Holotranscobalamin (cobalamin-saturated transcobalamin) was measured using a human holotranscobalamin ELISA kit (Abbexa Ltd., Cambridge, UK). Moreover, the difference between serum concentrations of total cobalamin and holotranscobalamin was calculated, which reflects the amount of cobalamin bound to transcobalamin I (haptocorrin), i.e., the inactive pool of serum cobalamin;
(3) Soluble transcobalamin receptor (CD320) was measured using a human CD320 ELISA kit (Cohesion Biosciences, London, UK). The assay was standardized in pg/mL and the values were converted to pmol/L assuming the molecular weight of 58 kDa [26,27] in order to enable the comparison with vitamin B12 and holotranscobalamin concentrations;
(4) Methylmalonic acid was measured using a methylmalonic acid ELISA kit (Abbexa Ltd., Cambridge, UK).

The ELISA tests were performed in duplicates, in series, following sample collection from all study participants (both cases and controls). The median period of frozen sample storage was 5 months (maximum 10 months) for both the study and the control groups. For each ELISA assay, the samples were defrosted on the day of testing, and all samples were tested on the same day using the kits with the same lot numbers. The standard curve was run on each microplate according to the manufacturer’s instructions. The ELISA testing was performed by researchers blind to the status of the study participants (study or control group). The intra-assay and inter-assay precision for the used ELISA kits was <10%, according to the manufacturers’ information. A microplate reader, BioTek Synergy ATX (Agilent Technologies Inc., Santa Clara, CA, USA), was used for the plates’ reading. The purchase of this equipment was supported by a grant from the Priority Research Area qLIFE under the Strategic Programme Excellence Initiative in Jagiellonian University (grant no. U1C/P04/NO/04.05).

### 2.4. Statistical Analysis

Categorical data were summarized as number (*n*) of patients and percentage of the respective group. Quantitative variables were summarized using mean ± standard deviation (SD; when normally distributed) or median (lower; upper quartile) (when non-normally distributed). The variables’ distributions were tested for normality with the Shapiro–Wilk’s test. The categorical variables were compared between groups with Pearson’s chi-squared test. The quantitative variables were compared between groups with a *t*-test or Mann–Whitney test, according to distribution. Moreover, logistic regression adjusted for important confounders (such as age or vitamin B12 intake) was used for the groups’ comparison, as indicated in detail in the Results section. The correlations were studied with Pearson or Spearman rank correlation coefficient, depending on the variables’ distributions. Linear regression was used as described in the Results section to assess if the selected correlations are affected by the confounders. All statistical tests were two-tailed and *p* < 0.05 was considered to indicate statistically significant results. Statistica 13 software package (Tibco Inc., Tulsa, OK, USA) was used for calculations.

## 3. Results

### 3.1. General Characteristics of the Studied Groups

The study included 80 patients, 39 girls and 41 boys, aged 1–18 years. Forty-one participants were diagnosed with FASD and thirty-nine were qualified as healthy controls (Table 1). In the FASD group, 19 patients (46.3%) were diagnosed with fetal alcohol syndrome (FAS), and 22 (53.7%) with alcohol neurobehavioral disorder–prenatal alcohol exposure (ND-PAE). Most patients with FASD were raised in foster and adoptive families. The studied group was characterized by a lower age, gestational weight, and weight and height at admission as compared to controls (Table 1). In logistic regression adjusted for age, neither weight (*p* = 0.2) nor height (*p* = 0.085) at admission differed significantly between the FASD and control group.

The FAS and ND-PAE subgroups did not differ in sex (*p* = 0.3) and age (*p* = 0.2). The gestational age (median 36 versus 39, respectively; *p* = 0.011) and gestational weight (median 2200 versus 2780 g; *p* = 0.011) were lower in patients with FAS. As expected, the FAS group was characterized by lower head circumference (median 2nd versus 25th percentile; *p* = 0.037), height (median 3rd–10th versus 25th–50th percentile; *p* < 0.001) and weight (median 3rd–10th versus 25th–50th percentile; *p* < 0.001) percentiles at admission, as well as having lower palpebral fissure length (median −2.88 versus −0.56 SD; *p* = 0.011) and increased philtrum smoothness (median 4 versus 1 points on Likert scale; *p* = 0.006) compared to ND-PAE. More patients diagnosed with FAS were living in adoptive families (*n* = 10; 52.6%) and fewer were in foster or institutional care (*n* = 9; 47.4%) as compared to those with ND-PAE (*n* = 3, 13.6% and *n* = 16, 72.7%, respectively; *p* = 0.017). All three patients with FASD raised in biological families were diagnosed with ND-PAE.

### 3.2. Vitamin B12 Intake and Concentrations in FASD and Control Groups

In simple analysis, the reported daily intake of vitamin B12 was higher in the FASD group than in healthy controls (Table 2). In logistic regression adjusted for age, the FASD and control groups still differed significantly in daily intake of vitamin B12 (OR for FASD: 1.82; 95% CI: 1.16–2.87; *p* = 0.008).

Nonetheless, we did not observe significant differences between the groups in serum concentrations of total cobalamin, holotranscobalamin, the difference between total cobalamin and holotranscobalamin, CD320 or methylmalonic acid (Table 2). Serum total cobalamin above the reference range was observed only in the FASD group (Table 2), while there were no results below the reference interval. The five patients with FASD with serum total cobalamin above the upper reference limit were characterized by a high difference between total cobalamin and holotranscobalamin; the median (Q1; Q3) was 802 (801; 830) in this group compared to 247 (157; 319) in the rest of the FASD group (*p* < 0.001), and this was the only statistically significant difference between these subgroups. Moreover, the serum concentrations of total cobalamin did not differ significantly between the FASD and control groups after adjustment for age and/or daily intake of vitamin B12.

### 3.3. The Associations of Vitamin B12 Intake with Other Studied Variables in FASD and Control Groups

In the control group, the daily intake of vitamin B12 was significantly higher in boys than in girls (3.31 ± 1.17 versus 2.37 ± 0.99 µg; *p* = 0.010). We did not observe any other associations with demographic or clinical characteristics of control subjects (age, sex, gestational age or weight, weight and height at admission; all *p* values > 0.2). Moreover, the daily intake of vitamin B12 did not correlate with the results of the laboratory tests associated with vitamin B12 status in the control group, i.e., serum total cobalamin (R = 0.06; *p* = 0.7), holotranscobalamin (R = 0.16; *p* = 0.3), total cobalamin−holotranscobalamin (R = 0.05; *p* = 0.8), methylmalonic acid (R = −0.19; *p* = 0.2) and CD320 (R = −0.08; *p* = 0.6).

In the FASD group, there were no sex-related differences in daily vitamin B12 intake (mean 3.33 ± 0.88 µg in boys versus 3.71 ± 1.14 µg in girls; *p* = 0.2). However, the daily intake of vitamin B12 positively correlated with head circumference (R = 0.45; *p* = 0.005) and weight (R = 0.38; *p* = 0.014) percentiles at admission. There were no significant differences regarding the intake of vitamin B12 between the patients raised in adoptive families versus foster/institutional care (mean 3.51 ± 0.80 and 3.45 ± 1.12 µg, respectively; *p* = 0.2), nor between patients diagnosed with FAS versus ND-PAE (mean 3.28 ± 1.09 and 3.70 ± 0.93 µg, respectively; *p* = 0.5). Considering laboratory results, we observed a significant positive correlation of vitamin B12 intake with serum holotranscobalamin levels (R = 0.37; *p* = 0.017).

**Figure 1:**
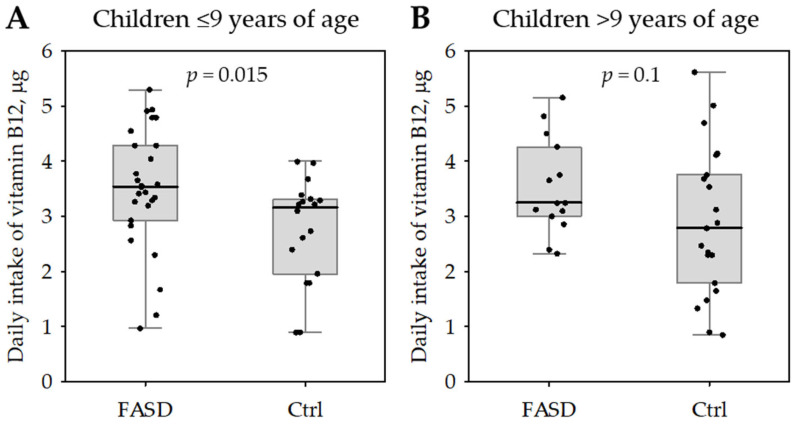
Daily intake of vitamin B12 among patients with fetal alcohol spectrum disorder (FASD) compared with healthy controls (Ctrl). The data are shown separately for children with ages below or equal to the median (9 years; (**A**)) and above the median (**B**). The data are shown as median (central bar), interquartile range (box), non-outlier range (whiskers) and raw data (points). The *p*-values for the comparisons are shown on the graphs.

When we stratified the whole studied group according to the median age (below/equal to versus above 9 years), only the children ≤ 9 years significantly differed in the daily intake of vitamin B12 (Figure 1).

### 3.4. The Associations of Serum Vitamin B12 Status with Other Studied Variables in FASD and Control Groups

In the control group, serum total cobalamin (R = −0.58; *p* < 0.001), total cobalamin−holotranscobalamin (R = −0.60; *p* < 0.001), methylmalonic acid (R = −0.61; *p* < 0.001) and CD320 (R = −0.42; *p* = 0.008) levels were all negatively correlated with age. No such correlation was observed for serum holotranscobalamin. Consequently, serum total cobalamin and methylmalonic acid levels negatively correlated with height and weight at admission, but there were no correlations with height or weight percentiles. There were no significant associations of the studied laboratory results with the sex of control subjects. We observed a positive correlation between serum concentrations of total cobalamin and methylmalonic acid (R = 0.53; *p* = 0.001); however, it became insignificant (*p* = 0.4) after adjustment for age. Furthermore, serum CD320 was positively correlated with the difference between serum total cobalamin and holotranscobalamin (R = 0.37; *p* = 0.033).

The studied laboratory results showed diverse associations in the FASD group. We did not observe any significant associations with age (R = −0.29; *p* = 0.08 for serum total cobalamin; R = −0.29; *p* = 0.07 for the difference between total cobalamin and holotranscobalamin; *p* > 0.4 for other results). The serum holotranscobalamin concentrations differed significantly between boys and girls (median 78.3 versus 120 pmol/L, respectively; *p* = 0.025). There were no significant associations with the diagnosis (FAS versus ND-PAE) or type of care (adoptive versus foster/institutional). Interestingly, gestational age correlated positively with serum CD320 (R = 0.52; *p* = 0.001) levels, and gestational weight correlated positively with both serum total cobalamin (R = 0.35; *p* = 0.041) and CD320 (R = 0.47; *p* = 0.003) levels. Moreover, there was a positive correlation between serum CD320 levels and palpebral fissure length (R = 0.48; *p* = 0.002).

**Figure 2:**
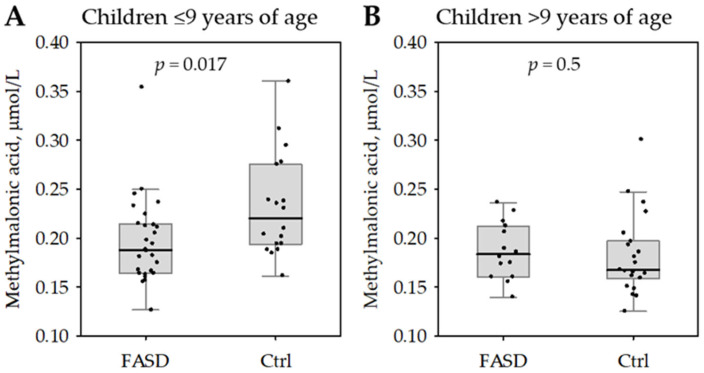
Serum concentrations of methylmalonic acid among patients with fetal alcohol spectrum disorder (FASD) compared with healthy controls (Ctrl). The data are shown separately for children with age below or equal to the median (9 years; (**A**)) and above the median (**B**). The data are shown as median (central bar), interquartile range (box), non-outlier range (whiskers) and row data (points). The *p*-values for the comparisons are shown on the graphs.

We observed significant differences in serum methylmalonic acid concentrations between the FASD and control group in the younger children (≤9 years of age) but not the older children (Figure 2). There were no similar observations regarding serum total cobalamin, holotranscobalamin, the difference between the two forms or CD320.

## 4. Discussion

Children with FASD are known to suffer from feeding disorders [28,29,30]. Food selectivity, picky eating, delay in acquisition of feeding competences and impaired regulation of appetite were described in this group of patients [28,29,30]. Moreover, children with FASD tend to snack and hide food. Werts et al. established that the diet of children with FASD was imbalanced and characterized by excessive sugar consumption [30]. Overall feeding behaviours may contribute to inappropriate intake of both micronutrients and macronutrients. Low intake of fibre, potassium, vitamin A, vitamin K, omega-3 fatty acids, choline, vitamin D and calcium were reported among patients with FASD [31,32]. Dietary patterns similar to the ones observed among patients with FASD are thought to lead to low serum cobalamin levels [33]. However, we observed a higher B12 intake among children with FASD as compared to the control group, which was an unexpected finding. In our sample, children with FASD were mostly raised in adoptive and foster families. Feeding a child and nurturing their needs is a very emotional process that influences one’s self-assessment as a parent [28]. Adoptive and foster parents experience increased parenting stress [29,30], and those raising children with FASD are at even higher risk of stress and anxiety [31]. Therefore, we speculate that some of the self-reported dietary questionnaires might have been affected by parental attitudes, with a tendency to show themselves in a favourable light. It has to be mentioned that, although the vitamin B12 intake was higher in the FASD group, it did not appear excessive as compared with population data [34,35]. On the other hand, we observed positive correlations between vitamin B12 intake and weight as well as head circumference percentile in the FASD group. As growth deficiency [36] and microcephaly [37] are key features in FASD and can be considered measures of FASD severity [38], this finding suggests implication for further research.

Increased (as compared to controls) intake of vitamin B12 in the FASD group was not associated with increased serum cobalamin levels. In our sample, the serum concentrations of cobalamin, holotranscobalamin and soluble transcobalamin receptor did not differ between the FASD group and the control group. This observation may result from the fact that vitamin B12 absorption is highly variable and depends on the exact dietary source and other factors [39]. The data on the relationship between vitamin B12 intake and the concentrations of cobalamin function biomarkers in the pediatric population are scarce [40]. However, Hay et al. documented that, among toddlers, an increase in vitamin B12 dietary intake was associated with an increase in holotranscobalamin, although a plateau was reached with consumption of approximately ~3 μg/day [41]. Although age variability in our FASD sample may influence the interpretation, mean vitamin B12 intake was above 3 μg/day. Therefore, the lack of differences between the groups might result from the fact that, in concordance with the results of Hay et al., a plateau in holotranscobalamin had been reached. In the control group, vitamin B12 intake did not correlate with any of the serum parameters. However, in the FASD group a significant positive correlation between vitamin B12 intake and serum holotranscobalamin was found. Considering no significant differences in holotranscobalamin concentrations between the groups, this finding requires careful interpretation. Published data indicate that only 4.4–16.9% of serum transcobalamin is saturated with cobalamin [42]. Holotranscobalamin is the essential fraction of serum cobalamin bound by the membrane receptor and taken up by body cells [43]. Therefore, our observations may suggest that in FASD there may be a tendency for more effective utilization of consumed vitamin B12.

Fetal alcohol spectrum disorder is a neurodevelopmental condition resulting from PAE. There are various hypotheses on how PAE influences the fetal brain [3]. Previously published data suggest that co-supplementation of cobalamin may mitigate the teratogenic influence of alcohol on fetal growth and morphological anomalies [44,45]. Xu et al. utilized a mouse model in an experiment which demonstrated that combined supplementation of folic acid and cobalamin between gestational days 1 to 16 elevated maternal serum levels of folic acid and cobalamin, decreased maternal serum levels of homocysteine and mitigated ethanol-induced prenatal mortality and growth delays in fetuses [44]. Breton-Lavrischee et al. used a CB57BL/6 mouse model and presented the idea that enhancing the maternal diet with folic acid, choline, betaine and vitamin B12 before conception and during gestation significantly diminished the occurrence and severity of alcohol-induced morphological anomalies [45]. Moreover, alcohol use in pregnancy might be a risk factor for B12 deficiency [46,47]. Adequate maternal cobalamin levels were correlated with an increased likelihood of children exhibiting superior motor, gross motor and receptive-language skills [48]. We therefore hypothesized that cobalamin metabolism and cobalamin status may differ between children/adolescents with PAE (diagnosed with FASD) and their non-exposed peers either due to unrevealed maternal cobalamin deficiency or as a compensating mechanism due to the potentially protective role of cobalamin. Yet, we did not document differences between the groups’ cobalamin and related markers. However, we observed significant negative correlations of serum total cobalamin, holotranscobalamin and the difference between the two forms with age in the control children (which is in line with previous observations in healthy children [25], while these associations were insignificant in patients with FASD. Contrarily, serum total cobalamin in the FASD group was positively correlated with gestational weight and holotranscobalamin differed between sexes. 

CD320 is a cellular holotranscobalamin receptor. The extracellular domain (soluble CD320) is found in plasma and other body fluids. Serum CD320 was observed to positively correlate with serum cobalamin and to bind a fraction of holotranscobalamin in serum [26,49]; however, its role has not been fully understood yet [50]. Increased sCD320 levels were documented in both serum and urine during pregnancy [49]; however, no data have been published regarding the pediatric population. In the present study, we observed that in the FASD group serum CD320 correlated positively with gestational age, gestational weight and palpebral fissure length, while it was negatively correlated with age in controls. This is an interesting finding because both palpebral fissure length and gestational weight are negatively correlated with FASD severity, and low gestational weight and shorter palpebral fissure are indicative of greater prenatal alcohol exposure [51,52]. Our results have to be interpreted with caution; however, this phenomenon should be explored in the future.

We observed that among patients with FASD, there is a higher chance of markedly increased cobalamin levels (i.e., above the age- and sex-related reference intervals) in comparison to controls. In the present study, 12% of studied children/adolescents with FASD presented with serum total cobalamin above the upper reference limit. In the studied children with FASD with high total cobalamin, we also observed a high difference between total cobalamin and holotranscobalamin, indicative of elevated concentrations of vitamin B12 bound to transcobalamin I (haptocorrin). We did not observe other significant differences between those children and the patients with FASD with normal serum cobalamin; in particular, no differences were observed in vitamin B12 dietary intake nor the serum concentrations of holotranscobalamin, which is considered an active fraction of the vitamin. This finding may suggest either increased production or decreased turnover of haptocorrin—the mechanisms previously described in diseases associated with high serum cobalamin [53,54]. However, unlike conditions such as cancer, kidney or liver diseases [54,55,56,57], in which high serum cobalamin was described, FASD is a neurodevelopmental condition with scarce systemic manifestations. Indeed, increased serum levels of vitamin B12 have been previously associated with various neurodevelopmental conditions as compared to healthy controls [14,15]. Hope et al. indicated that patients with neurodevelopmental disorders present with a markedly elevated serum level of vitamin B12 compared to healthy controls [14]. These findings align with a recent study indicating that elevated levels of vitamin B12 during pregnancy are correlated with a two-fold rise in the risk of autism spectrum disorder (ASD) in the offspring, and that both excessive and insufficient intake of vitamin B12 are linked with such heightened risk [58]. Furthermore, in another study a non-significant tendency towards elevated serum vitamin B12 was also observed in ASD [59]. Our present results are also consistent with previous findings of Dylag et al., who identified elevated levels of vitamin B12 in 8% of individuals with FASDs, with a tendency towards FAS over ND-PAE (although the difference between FAS and ND-PAE was not statistically significant, possibly due to the relatively small sample size). These previous results suggest that a more severe clinical presentation (FAS) is associated with increased serum vitamin B12 [16]. In the present study, we have not confirmed such a relationship, which also may result from small sample size. In agreement with the authors of the aforementioned studies, we struggle to determine which mechanisms can be responsible for the observed phenomenon. As the role of cobalamin in myelination is essential [60] and delayed or impaired myelination was documented in FASD [4,5,6,7,8], we would like to emphasize that the incidence of high cobalamin concentrations in this group of patients should be investigated in the future.

This is the first attempt to characterize cobalamin status among patients with FASD. Our study has several limitations, namely, the relatively small sample size, the use of parental self-report for the assessment of dietary intake of vitamin B12, and the use of only ELISA tests to measure serum concentrations of holotranscobalamin, CD320 and methylmalonic acid. Considering these limitations, our results should be interpreted with caution. Nonetheless, this study provides a comprehensive analysis of serum cobalamin and the related laboratory markers. We speculate that the age heterogeneity of our sample may influence our results. We suggest that particular emphasis should be made to investigate the characteristics of cobalamin metabolism in younger children, as the reminiscences of the prenatal period may be more evident in this group.

## 5. Conclusions

The present study aimed to characterize cobalamin status among patients with FASD. Our results indicate that patients diagnosed with FASD are not at risk of cobalamin deficiency. Contrarily, a subgroup of patients diagnosed with FASD presented with increased blood levels of total cobalamin, and patients with FASD had higher average daily dietary intake of vitamin B12 as compared to controls. Moreover, the studied laboratory markers showed different correlations with demographic and clinical factors in cases and controls, suggesting an association between vitamin B12 status in FASD and prenatal development. Further research is required to explain the mechanisms underlying the observed phenomena.

## Figures and Tables

**Table 1:** Characteristics of participants according to the group at the time of admission. Quantitative data are shown as arithmetic mean ± standard deviation or median (lower; upper quartile). FASD—fetal alcohol spectrum disorder.

Characteristic	FASD (*n* = 41)	Controls (*n* = 39)	*p*-Value
Age, years	8.2 ± 4.0	10.0 ± 4.0	0.028
Male sex, *n* (%)	22 (53.7)	19 (48.7)	0.7
Diagnosis: FAS/ND-PAE, *n* (%)	19 (46.3)/22 (53.7)	-	-
Institutional care, *n* (%)	2 (4.9)	-	-
Foster family, *n* (%)	23 (56.1)	-	-
Adoptive family, *n* (%)	13 (31.7)	-	-
Biological family, *n* (%)	3 (7.3)	-	-
Preterm birth, *n*/valid *n* * (%)	15/38 (39.5)	3/18 (16.7)	0.088
Gestational age, weeks	38 (35; 39)	38 (38; 40)	0.3
Gestational weight, g	2515 (2030; 3150)	3100 (3000; 3300)	0.032
Weight, kg	21.8 (16.9; 32.8)	33.2 (21.9; 49.6)	0.006
Weight < 10th percentile, *n* (%)	18 (43.9%)	3 (7.7)	<0.001
Height, m	120 (104; 142)	141 (124; 159)	0.004
Height < 10th percentile, *n* (%)	21 (51.2)	5 (12.8)	<0.001
Head circumference, cm	50 (49; 51)	NA	-
Head circumference, percentile	10 (<3; 25)	NA	-
Palpebral fissure length, SD	−1.98 (−3.09; 0.11)	NA	-
Philtrum smoothness, Likert scale	4 (3; 4)	NA	-

* Valid *n*—number of participants with available data; NA—not available.

**Table 2:** Daily intake of vitamin B12 and the results of laboratory tests related to vitamin B12 homeostasis according to the group. Quantitative data are shown as arithmetic mean ± standard deviation or median (lower; upper quartile). FASD—fetal alcohol spectrum disorder.

Characteristic	FASD (*n* = 41)	Controls (*n* = 39)	*p*-Value
Daily intake of vitamin B12, µg	3.51 ± 1.02	2.83 ± 1.17	0.008
Total cobalamin, pmol/L	396 (304; 557)	346 (287; 446)	0.1
Total cobalamin above the upper reference limit, *n* (%) *	5 (12.2)	0	0.024
Holotranscobalamin, pmol/L	94.1 (72.7; 132.5)	101.5 (82.1; 126.2)	0.6
Total cobalamin−holotranscobalamin, pmol/L	269 (189; 466)	249 (157; 319)	0.3
Soluble transcobalamin receptor (CD320), pmol/L	121 (8.20; 267)	108 (42.9; 345)	0.5
Methylmalonic acid, µmol/L	0.19 (0.16; 0.21)	0.19 (0.17; 0.24)	0.4

* Reference interval according to Bailey et al. [25].

## Data Availability

The data presented in this study are available on request from the corresponding author due to privacy restrictions.
